# Complex Recombination Patterns Arising during Geminivirus Coinfections Preserve and Demarcate Biologically Important Intra-Genome Interaction Networks

**DOI:** 10.1371/journal.ppat.1002203

**Published:** 2011-09-15

**Authors:** Darren P. Martin, Pierre Lefeuvre, Arvind Varsani, Murielle Hoareau, Jean-Yves Semegni, Betty Dijoux, Claire Vincent, Bernard Reynaud, Jean-Michel Lett

**Affiliations:** 1 Computational Biology Group, Institute of Infectious Diseases and Molecular Medicine, University of Cape Town, Observatory, South Africa; 2 CIRAD, UMR 53 PVBMT CIRAD-Université de la Réunion, Pôle de Protection des Plantes, Ligne Paradis, Saint Pierre, La Réunion, France; 3 School of Biological Sciences, University of Canterbury, Christchurch, New Zealand; 4 Biomolecular Interaction Centre, University of Canterbury, Christchurch, New Zealand; 5 Electron Microscope Unit, University of Cape Town, Rondebosch, Cape Town, South Africa; University of Kentucky, United States of America

## Abstract

Genetic recombination is an important process during the evolution of many virus species and occurs particularly frequently amongst begomoviruses in the single stranded DNA virus family, *Geminiviridae*. As in many other recombining viruses it is apparent that non-random recombination breakpoint distributions observable within begomovirus genomes sampled from nature are the product of variations both in basal recombination rates across genomes and in the over-all viability of different recombinant genomes. Whereas factors influencing basal recombination rates might include local degrees of sequence similarity between recombining genomes, nucleic acid secondary structures and genomic sensitivity to nuclease attack or breakage, the viability of recombinant genomes could be influenced by the degree to which their co-evolved protein-protein and protein-nucleotide and nucleotide-nucleotide interactions are disreputable by recombination. Here we investigate patterns of recombination that occur over 120 day long experimental infections of tomato plants with the begomoviruses *Tomato yellow leaf curl virus* and *Tomato leaf curl Comoros virus*. We show that patterns of sequence exchange between these viruses can be extraordinarily complex and present clear evidence that factors such as local degrees of sequence similarity but not genomic secondary structure strongly influence where recombination breakpoints occur. It is also apparent from our experiment that over-all patterns of recombination are strongly influenced by selection against individual recombinants displaying disrupted intra-genomic interactions such as those required for proper protein and nucleic acid folding. Crucially, we find that selection favoring the preservation of co-evolved longer-range protein-protein and protein DNA interactions is so strong that its imprint can even be used to identify the exact sequence tracts involved in these interactions.

## Author Summary

Genetic recombination between viruses is a form of parasexual reproduction during which two parental viruses each contribute genetic information to an offspring, or recombinant, virus. Unlike with sexual reproduction, however, recombination in viruses can even involve the transfer of sequences between the members of distantly related species. When parental genomes are very distantly related, it is anticipated that recombination between them runs the risk of producing defective offspring. The reason for this is that the interactions between different parts of genomes and the proteins they encode (such as between different viral proteins or between viral proteins and the virus genomic DNA or RNA) often depend on particular co-evolved binding sites that recognize one another. When in a recombinant genome the partners in a binding site pair are each inherited from different parents there is a possibility that they will not interact with one another properly. Here we examine recombinant genomes arising during experimental mixed infections of two distantly related viruses to detect evidence that intra-genome interaction networks are broadly preserved in these genomes. We show this preservation is so strict that patterns of recombination in these viruses can even be used to identify the interacting regions within their genomes.

## Introduction

Although variations in the basal mechanistic predispositions of different regions of nucleic acid molecules to recombine is certainly a primary determinant of recombination patterns detectable within some viral genomes [1]–[5], it is becoming increasingly apparent that an important secondary determinant is natural selection [6]–[8]. Functional analyses of recombinant genes [9], [10] and genomes [11]–[13] has indicated that a large proportion (and possibly the vast majority) of recombination events between distantly related genomes probably yield defective progeny.

Analyses of bacterial [14]–[16] and viral recombination [6], [12], [13], [17] and protein engineering studies utilising DNA-shuffling methodologies [9], [10], [15], [18] have indicated that the probability of a given recombination event being deleterious depends on the modularity of the specific gene(s) or sub-gene module(s) that are transferred and tends to increase with decreasing parental sequence relatedness. This effect is caused, at least in part, by the tendency of recombination to disrupt the networks of genome encoded sequence specific interactions that underpin the biology of all organisms. Examples of such encoded interactions might include inter-amino-acid contacts that determine and maintain proper protein folding, Watson-Crick base pairing within functionally meaningful secondary structures that form in single stranded DNA (ssDNA) and RNA molecules, and DNA and amino acid sequence motifs that mediate protein-protein, protein-DNA and protein-RNA interactions. It might therefore be expected that the survival of recombinant genomes under natural conditions will be largely dependent on how severely recombination has impacted such interactions.

To a degree, accurate *a priori* inference of the approximate fitness costs of recombination events has already been achieved within the context of individual proteins for which inter-amino acid interaction networks can be inferred from atomic resolution 3D structural information [9], [19]. Such approximations have been successfully used to increase the efficiency of DNA shuffling based protein engineering approaches [20]–[22]. However, replicating these successes at a whole-organism scale to, for example, assess the risks of novel pathogens emerging via recombination between different viruses co-infecting a particular host species, would require high resolution information on multiple genome-wide interaction networks – information that is not available for even the most well studied virus species. Also, rather than trying to use such interaction networks to explain recombination patterns, it might actually be more productive to use recombination patterns to infer the interaction networks. Given that recombination patterns are probably strongly influenced by these networks, it is reasonable to suspect that recombination patterns might encode information on their architectures.

We investigated this possibility in experimentally constituted mixed begomovirus infections. Species in the genus *Begomovirus* (Family *Geminiviridae*) are ideal test subjects in this regard not only because the emergence of numerous begomoviral crop pathogens has been attributed to natural inter-species recombination [23]–[27], but also because begomovirus genomes contain various well characterised intra-protein [28], [29], inter-protein [30] and protein-DNA interactions [31]–[34]. Using a genome-wide association approach and a variety of permutation tests to analyse data from controlled evolution experiments we demonstrate how recombination patterns can be used to: (1) identify mechanistic causes of variations in basal recombination rates across begomovirus genomes, (2) identify host adaptive nucleotide polymorphisms, (3) identify the various ways in which recombination can disrupted intra-genome interactions, and (4) indirectly visualise long-range intra-genome interaction networks.

## Materials and Methods

### Virus sources

Agroinfectious clones of *Tomato yellow leaf curl virus* - Mild [Reunion:2002] (AJ865337, referred to here as TYX) and *Tomato leaf curl Comoros virus* – [Mayotte:Dembeni:2003] (AJ86539, referred to here as TOX) are described in Urbino et al. [35]. Relative to one-another the two viruses display nucleotide polymorphisms at 491 genome sites (i.e. at ∼17.8% of their total genomic nucleotides) and differ at an additional 13 sites displaying between 1 and 14 nucleotide insertions/deletions.

### Plant inoculation

A total of 89 seven day old tomato seedlings (Farmer variety, Known-you Seed) were co-agroinoculated with both TYX and TOX and were checked fourteen days later for evidence of co-infection using specific PCR primers (TYX forward: CCCAATTTTCAAGGATATG; TYX reverse: GCGCTTCCAAATAAAATTGC; TOX forward: AGGCTTTCAGGGGTGCA; TOX reverse: GTCGTTTCAGCATCAAAGC). Out of 33 successfully co-infected plants, ten randomly selected plants were grown for a total of four months before leaf samples were collected and stored as previously described by Bos [36].

### Genome sequencing

Total DNA was extracted from leaf samples using the DNeasy Plant miniprep kit (Qiagen) according to the manufacturer's instructions. Circular viral DNA molecules were amplified using the TempliPhi Kit (GE Healthcare) as described by Inoue-Nagata et al., [37]. Full genomes were cloned into either pGEM-3Zf or pGEM-7Zf (Promega, USA) vectors at their *Bam*HI restriction sites. A total of 362 complete DNA-A-like components from the ten plants (Table S1) were cloned and sequenced using the Macrogen Inc. sequencing service (Korea).

### Recombination detection

Identification of potential recombination breakpoint regions and the origins of recombinant fragments was carried out for each of the cloned sequences using the computer program RDP3 ([38]; available from http://darwin.uvigo.es/rdp/rdp.html) which was set up to perform direct nucleotide-by-nucleotide comparisons of potential recombinants and a pair of known parental sequences (in this case the input TYX and TOX sequences). A breakpoint map was compiled denoting the positions of all clearly identifiable unique recombination breakpoints (breakpoints falling at identical sites were considered different only if they were observed in viruses cloned from different plants).

### Detection of recombination breakpoint hot- and cold-spots

Breakpoint density plots were then constructed from this map as described in [39]. Briefly, 200, 100, 75 or 50-nt windows were moved one nucleotide at a time along the length of the map and at each window position counts of all clearly identifiable unique recombination breakpoints falling within the window were plotted at the central window position. Significant clustering of breakpoint positions within each window was tested using the recombination hot-spot permutation test described in Heath et al. [39].

### Determining the influence of local sequence similarity on recombination breakpoint frequencies

To test whether local degrees of shared sequence similarity between TYX and TOX influenced where recombination occurred between their genomes, we sorted the observed recombination breakpoint positions based on the numbers of contiguous identical nucleotides shared between the two genomes at the sites where the breakpoints were detected. We used a simple permutation test to determine whether the observed frequency of recombination breakpoints at each of these site categories was significantly different from those expected at these sites assuming that recombination breakpoints were randomly distributed and uninfluenced by local degrees of sequence similarity (see Protocol S1 for details).

### Determining the influence of predicted local ssDNA structure on recombination breakpoint frequencies

Given that the secondary structures of the TYX and TOX genomes are unknown, we inferred an ensemble of near minimum free energy (MFE) folds for each genome using the program UNAFold version 3.8 [40], with sequences treated as circular ssDNA, the annealing temperature set to 25°C and sodium and magnesium concentrations respectively set to 1M and 0M. UNAFold was additionally set-up to only allow Watson-Crick (GC, AU) and Wobble (GU) base pairings. From these we produced, for each parental genome, a unified fold by scoring each predicted base-pairing interaction as the proportion of times that interaction was found within the 22 and 19 near minimum free energy (MFE) predicted folds of the aligned TYX and TOX genomes respectively.

Previous investigations into the impact of ssDNA secondary structure on begomovirus recombination frequencies have indicated that recombination breakpoints tend to occur at genome sites where one parental genome has a stable secondary structure and the other does not [26]. We therefore tested for evidence of increased recombination breakpoint frequencies within four separate sets of sites: (1) those at which nucleotides were predicted to be involved in base-pairing within either the TYX or TOX genomes, (2) those at which nucleotides were predicted to be base paired in the TYX but not the TOX genome and (3) those at which nucleotides were predicted to be base paired in the TOX but not the TYX genome. In order to avoid the biasing influences of the presumed (and strongly predicted) stem-loop sequence at the virion strand origin of replication (a known major recombination hotspot) we excluded from these sets all nucleotides falling within the 41 100% identical nucleotides shared by TYX and TOX around this region.

Evidence of breakpoint clustering within the various structured nucleotide sets (i.e. nucleotides inferred to be paired in both/either/one-or-the-other parents) was tested using a slightly modified version of the permutation tests described in Simon-Loriere et al. [5] and Lefeuvre et al. [41] (see Protocol S1 for details). Briefly, for the four different sets of sites tested, this involved dividing sites within the alignment into those falling within the test-set and those falling outside the test set, and then testing for significantly increased recombination breakpoint frequencies within the test set.

### Detection of selection acting against dysfunctional recombinants

The fundamental aim of our study was to determine the influence of encoded intra-genome interaction networks on inter-species recombination patterns arising during co-infections. To achieve this we sought to test for evidence of selection acting on recombinants in such a way as to maintain the stability of various hypothetical interaction networks including those occurring between: (1) amino acids within expressed proteins, (2) viral nucleotides within predicted genomic DNA secondary structures, and (3) viral genomic regions (e.g. long-range interactions occurring between either different viral proteins or between virus proteins and viral DNA). Specifically, we attempted to statistically test whether, and to what extent, maintenance of these various interactions influences the recombination patterns that arise during co-infections. An important assumption that we made throughout is that recombinant viruses sampled from these co-infections (or at least a subset of these viruses) have been acted upon by selection and are reasonably fit members of the populations from which they were sampled. However, considering both the high evolution rates of begomoviruses [42]–[44] and the probable quasi-species and/or genetic complementation dynamics at play during infections involving genetically diverse virus populations [45]–[47], we expected this assumption to be somewhat violated for many of the low frequency genetic variants that were discovered during extensive sampling of genomes within the various co-infections analysed. In order to both limit and quantify potential biases caused by the sampling of low fitness recombinant variants, we analysed on the one hand the entire set of sampled recombinants – called our FULL dataset – and on the other only those presumably viable and reasonably fit recombinants that were sampled multiple times from individual populations – called our FIT dataset. We must stress here that even when recombinants were sampled multiple times from infected plants the possibility remained that these might still have been defective and that their higher prevalence could have been the result of either random drift or complementation by fit genomes. However, rather than biasing analyses of the FIT dataset in favour of detecting selection against defective genomes, inadvertent inclusion of subtly defective recombinants in the FIT dataset would have degraded our ability to use this dataset to detect selection against defective recombinants.

### Protein folding disruption tests

Probably as a consequence of the fact that recombination breakpoints that occur within protein coding sequences can potentially disrupt protein folding, it has been noted that recombination breakpoints detectable in viruses sampled from nature tend to cluster either within non-coding regions or at the boundaries of genes [7], [41], [48], [49]. We firstly tested whether recombination breakpoints within the recombinants arising during co-infections displayed a tendency to fall outside coding regions or on the periphery of genes using the same tests described in Lefeuvre et al. [41] (implemented in RDP3). We then applied the SCHEMA based tests described in Lefeuvre et al. [8] (also implemented in RDP3; [9]) to determine whether recombination breakpoints falling within the genome regions encoding the structurally modelled portions of Rep (the 118 N-terminal amino acids; [28]) and CP (196 amino acids; [29]) were less disruptive of protein folding than was expected by chance. For full details of these tests see Protocol S1.

### ssDNA folding disruption tests

In the same way that recombination has the potential to disrupt protein folding, it could potentially disrupt ssDNA folding. We used two separate permutation tests of ssDNA folding disruption to determine whether there was any evidence of recombinant sequences displaying significantly lower degrees of estimated ssDNA folding perturbation than that observed in randomly generated recombinant sequences. The permuted datasets used in both tests were identical. For each of 50 unique recombinant sequences we produced 100 recombinants with randomly placed breakpoints containing (1) the same number of recombination breakpoints as the real recombinant, (2) the same number of TOX and TYX derived polymorphisms as the real recombinant, and (3) the same numbers of polymorphic sites between recombination breakpoints as the real recombinant. These simulated datasets were compared to the real one using (1) a test of global fold stability comparing MFE values and (2) a SCHEMA-like test very similar to that devised in Voigt et al. [9] for comparing conservation of nucleotide pairing in recombinant genomes (see Protocol S1 for details). In both ssDNA folding disruption tests we predicted for every recombinant (both real and simulated) an ensemble of ssDNA folds displaying free energies close to that of the MFE fold. As before the ssDNA/RNA folding program UNAFold [40] was used throughout with the same settings as those mentioned above.

### Association test to identify long-range intra-genome interactions

We sought to determine whether there was a signal of recombination having preserved known long-range intra genome interactions such as those occurring between: (1) the DNA binding domain of Rep and iterated Rep binding sites within the C1 proximal side of the intergenic region (a DNA-protein interaction; [31], [33], [34]), (2) the V1 promoter and the transcription activator protein (TrAP; encoded by the C2 open reading frame (ORF); another DNA-protein interaction; [32]), (3) Rep and the replication enhancer protein (REn) (a protein-protein interaction; [30]) and (4) Rep and the coat protein [50].

However, rather than directly testing whether these known long-range begomovirus intra-genome interactions were preserved in the recombinants arising during our experiments, we considered the problem from an alternative perspective. We hypothesised that if selection within our experiment favoured the survival of recombinants in which co-evolved long-range intra-genome interactions remained intact, then interacting sites within the genome should preferentially be inherited in pairs from one parent or the other. We were specifically interested in whether blindly examining all possible pairwise associations between sites would identify these known interactions with a high degree of statistical confidence.

To achieve this we designed a simple Chi-square based permutation test capable of detecting potential associations between pairs of polymorphic nucleotide sites within TYX-TOX recombinants (see Figure S1). Briefly, this involved counting, for all possible pairs of polymorphic nucleotide sites (i, j; TYX and TOX differ at 491 sites meaning that “i” and “j” are integers between 1 and 491 and that there are (491×490)/2 = 120295 possible polymorphic nucleotide site pairs), the numbers of times the four different possible combinations of TYX and TOX derived sites (TYX_i_-TYX_j_, TYX_i_-TOX_j_, TOX_i_-TYX_j_ and TOX_i_-TOX_j_ respectively encoded as site-pair categories 1, 2, 3 and 4) were observed across all of the analyzed recombinants. For each of the 120295 site pairs these four numbers were used to calculate a 2x2 Chi-square value that was used as a primary indicator of possible associations between the site pairs – whereas pairs of sites yielding low Chi-square values are more likely to have been independently inherited, the inheritance of site-pairs yielding high Chi-square values is more likely to have been non-independent. Non-independence could be due to close physical linkage within the genome or, if it occurs between distantly separated sites, it might be due to the need to maintain co-evolved encoded interactions between the sites.

In each of 10 000 permuted datasets, the experimentally observed recombination events were reconstructed from the parental sequences so as to maintain the numbers of polymorphic nucleotides separating observed breakpoint positions (i.e. maintaining the degree of polymorphic nucleotide shuffling) observed in the real recombinants but with the actual genomic positions of recombination breakpoints randomly shuffled. Sets of 120295 2x2 association tables were then determined for each of these 10 000 datasets. Instances when a site pair in the real dataset had an associated Chi-square value higher than 95% of the corresponding values in the 10 000 permuted datasets were interpreted as being indicative of there being a potential interaction between the sites. These potentially interacting site-pairs were then shortlisted and subjected to second set of tests aimed at classifying the exact natures of their associations.

These secondary association tests were again permutation based rank tests but, rather than being based on the over-all patterns of values in the 2x2 tables (as was the case with the first Chi-square based test), they were based on the magnitudes of individual values within the 2x2 tables formulated for the real dataset relative to values in corresponding tables from the permutation datasets. For example, with the table representing the polymorphic sites 1 (site 22 in TYX) and 9 (site 52 in TYX) there are 6 instances where recombinants had both sites from TOX, 43 instances where recombinants had both from TYX, 1 where site 1 was from TOX and site 9 was from TYX, 0 instances where site 1 was from TYX and site 9 was from TOX. The Chi value associated with this table is 41.88 – a number which is larger than 7 793/10 000 of the Chi values determined for the same site pair in the permuted datasets (i.e. the sites do not display an association that cannot be accounted for by their close physical linkage). Out of 10 000 permuted datasets, 4 104 had more than 6 instances where both sites at positions 1 and 9 were from TOX and 1 589 had more than 43 instances where both sites were from TYX. Thus assuming (1) random recombination, and (2) no selection on either individual sites or pairs of sites, the *p*-value for the test of whether both sites are not preferentially derived from TOX  =  0.4104 (4104/10 000) and the *p*-value for the test of whether both sites are not preferentially derived from TYX  =  0.1589.

Here, however, we were specifically most interested in identifying lower than expected frequencies of (1) TOX derived polymorphisms at the first site and TYX derived polymorphisms at the second (i.e. site pair category 2), (2) TYX derived polymorphisms at the first site and TOX derived polymorphisms at the other (i.e. site-pair category 3), and (3) combinations of (1) and (2) (i.e. site pair categories 2+3).

Finally, we identified potentially interacting site pairs as those at which (1) the Chi-square based test indicated a potential interaction with an associated permutation *p*-value <0.05 and (2) where mixtures of TYX and TOX derived polymorphisms at either or both of the sites occurred less frequently in the real dataset than in >95% of the permuted ones.

## Results and Discussion

### Complex recombinants frequently arise during TYX - TOX co-infections

**Figure 1:**
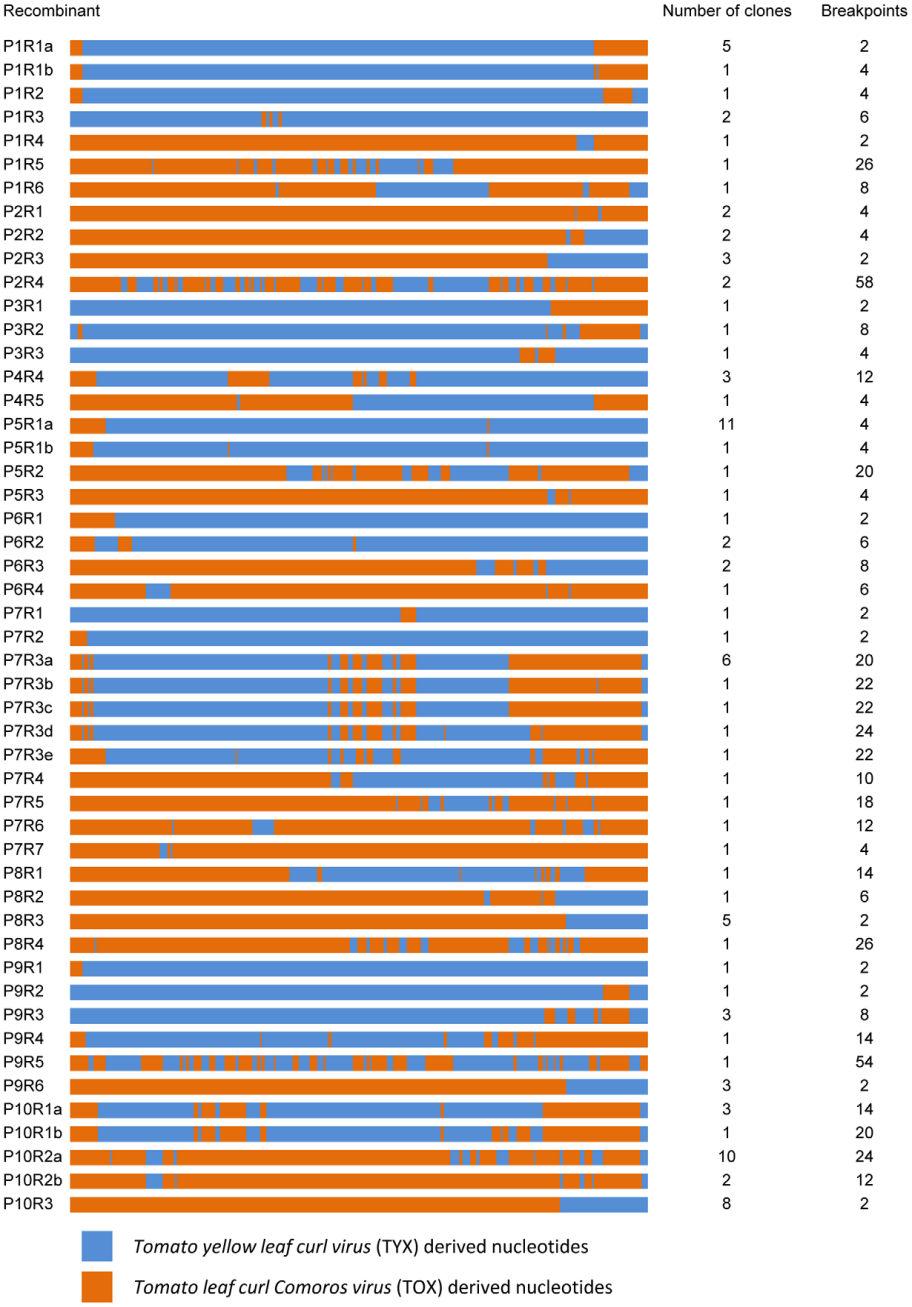
Unique recombinant genome mosaics arising during mixed *Tomato yellow leaf curl virus* (TYX) and *Tomato leaf curl Comoros virus* (TOX) infections. Each line represents one mosaic genome linearized at the virion strand origin of replication. The virus names to the left are in the form P[plant number]R[recombinant number]. Some recombinants are clearly minor variants of the same mosaic structure and are labelled a, b, c etc. Numbers of clones and breakpoints for each unique recombinant sequence are specified on the right of the figure. Eighteen of the 50 mosaics were observed in multiple clones from the same plant and are therefore probably not defective: These form the basis of our FIT dataset.

Of the 362 sequences obtained from ten independent four month old TYX-TOX tomato co-infections, 106 (∼ 29%) displayed evidence of having undergone one or more recombination events. Among these 106 were 50 unique recombinant genotypes (Figure 1), 18 of which were sampled multiple times and were therefore probably reasonably fit. The 32 recombinant genotypes for which only single genomes were sampled, were potentially minor population members in their respective plants and were therefore of uncertain viability (if these genomes had arisen immediately prior to sampling they would have avoided the influences of natural selection).

Within the 50 unique recombinant genotypes we collectively identified 452 clearly identifiable unique recombination breakpoints implying a mean of 9.04 detectable recombination events per recombinant sequence. Although complex recombinant genomes with up to 18 recombination breakpoints have been observed previously in maize streak virus recombination experiments [51], many of the recombinants that we observed in our experiment are extraordinarily complex (for example see recombinants P2R4 and P9R5 in Figure 1), containing as many as 58 detectable recombination breakpoints.

Interestingly, across the ten co-infection plants studied, the genetic distances between the recombinant genomes and their parents were not randomly distributed (Chi-square test *p*-value = 4.66.10^−16^). Whereas in six plants, the recombinant sequences were most similar to the TYX parent, in three plants, they were most similar to the TOX parent (Table S1). Only one plant presented viruses with balanced similarity to both parents. These differences between plants possibly indicate an experimental bias where stochastic differences in the initial numbers of TYX and TOX genomes co-infecting the plants eventually yielded recombinants carrying mostly TYX or TOX derived polymorphisms. Whereas in previously described geminivirus recombination experiments [26], [51], [52] independent replicated experiments appeared to deterministically yield very similar recombinant genomes, the widely varying outcomes observed in our experiment most likely reflect the complexity of the fitness landscape encountered within tomato by TYX, TOX and their recombinant progeny. In further analyses, we decided to pool viruses from different plants to improve the statistical power of the tests we performed. It should be borne in mind, however, that in so doing we ignored the potentially confounding factors underlying the over-all differences between recombinant virus populations arising in different plants.

### Identification of potentially host-adaptive polymorphisms

**Figure 2:**
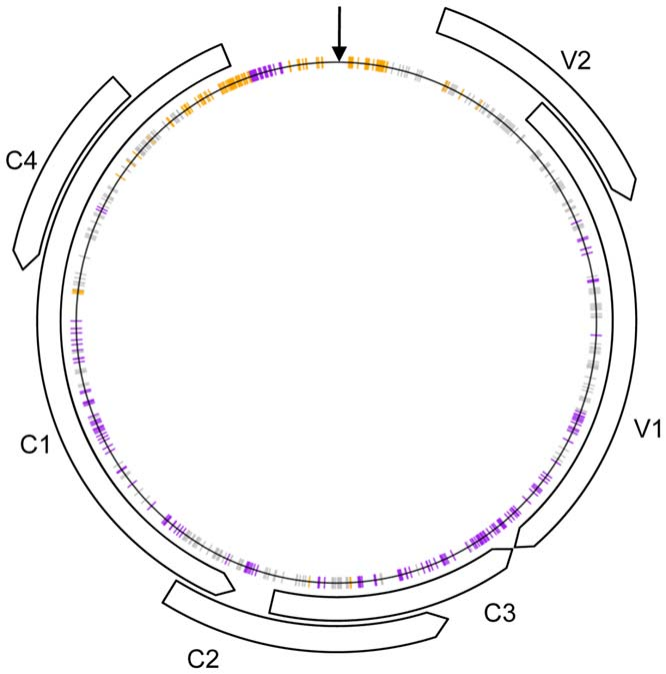
Schematic representation of the recombinant genomes indicating nucleotide positions at which more than expected *Tomato yellow leaf curl virus* (TYX; orange) or *Tomato leaf curl Comoros virus* (TOX; purple) derived nucleotides are found (*p*-value<0.001) under the assumption of random recombination. Polymorphic nucleotide sites indicated in grey represent those sites at which no preference for TYX or TOX derived nucleotides is evident. The positions of various open reading frames and the origin of replication (V-Ori) are indicated with arrows.

Within the recombinant genomes, of the 491 polymorphic nucleotide sites differentiating TYX and TOX, 121 (24.6%) almost exclusively situated within the intergenic region (IR) and the region of C1 encoding the Rep catalytic domain (Figure 2), were preferentially derived (*p*<0.001) from TYX. TOX derived nucleotides were significantly more common (*p*<0.001) at 192 sites (39.1%), mostly spread throughout the V1, C1, C2 and C3 ORFs (Figure 2). This indicates that both parental genomes possibly contained particular polymorphisms that performed better within the recombinants in tomato than those derived from the other parent.

The IR is the origin of virion and complementary strand replication in begomoviruses and is also involved in the regulation of gene expression. That this region is predominantly derived from TYX suggests that the TYX IR sequences are better adapted than their corresponding TOX analogues when it comes to productively interacting with the DNA synthesis and transcription machinery of tomato cells. It would be interesting to experimentally test this hypothesis to determine whether apparent adaptations within the TYX IR also provide a survival advantage either in other tomato genotypes or other begomovirus host species.

### Experimental recombination breakpoint patterns are non-random and largely mirror those seen in nature

**Figure 3:**
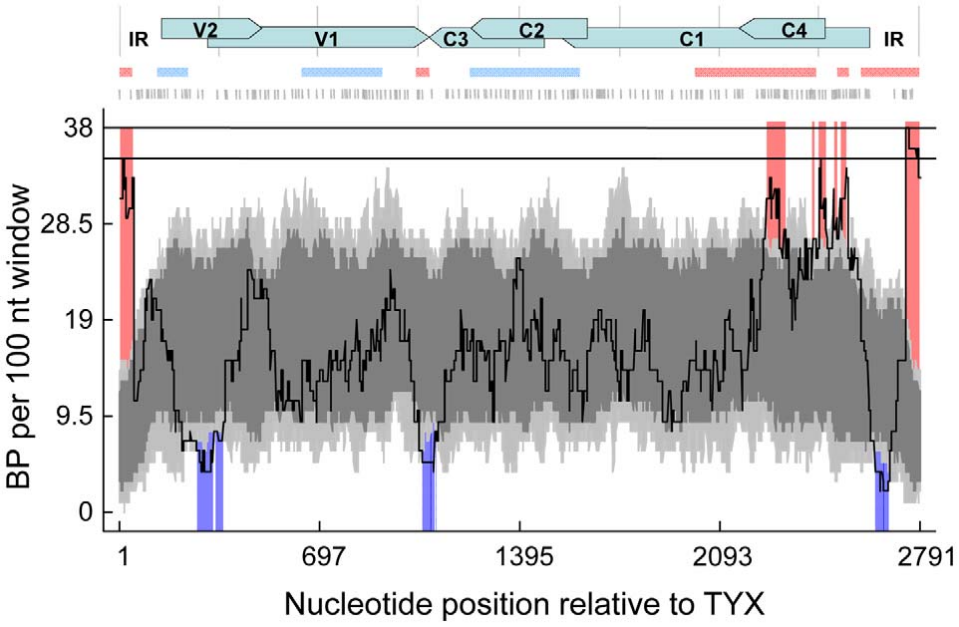
The distribution of 452 unique recombination breakpoints detected within the 50 unique recombinant sequences emerging during *Tomato yellow leaf curl virus* (TYX) and *Tomato leaf curl Comoros virus* (TOX) co-infections. All detectable unique breakpoint positions are indicated by small vertical lines at the top of the graph. A 100 nucleotide window was moved along the alignment one nucleotide at a time and the number of breakpoints detected within the window region was counted and plotted (solid line). The upper and lower broken lines respectively indicate 99% and 95% confidence thresholds for globally significant breakpoint clusters. Light and dark grey areas respectively indicate the expected 99 and 95 percentiles of expected recombination breakpoint clustering assuming random recombination. Whereas red areas indicate recombination hot-spots, blue areas represent recombination cold-spots. The positions of open reading frames (ORFs; horizontal arrows) and intergenic regions (IR) are represented on the top of the graph. Pink and blue horizontal lines beneath the ORF map respectively delineate the positions of recombination hot and cold spots detectable within begomovirus genomes sampled from nature (from Lefeuvre et al. [8]).

To visualize the overall distribution of the 452 unique recombination breakpoints detectable within the recombinant genomes that arose during the co-infections, we plotted them on a density map and tested this for evidence of recombination hot- and cold-spots. This analysis revealed that the distribution of breakpoints was non-random, with there being clear evidence of statistically significant recombination hot- and cold-spots (Figure 3 and Figure S2). Irrespective of the window size used in these permutation based recombination hot- and cold-spot tests, a single highly significant recombination hot-spot was apparent in the IR around the origin of virion strand replication. Depending on the window sizes used, either one or two highly significant breakpoint hotspots were also detectable within the C1 ORF on either side of the region overlapping the C4 ORF. Besides these recombination hot-spots we also detected cold-spots within the IR (upstream of the C1 start codon), at the overlapping part of the V1 and V2 ORFs and at the interface between the V1 and C3 ORFs (see Figure S2).

Consistent with these results, previous experimental studies and analyses of recombinant genomes sampled from nature have revealed the presence of recombination hot-spots within geminivirus IR and complementary sense gene sequences and cold-spots within virion sense gene sequences [53], [54]. It has been proposed that complementary sense gene transcription, which occurs in the opposite direction to rolling circle replication, may increase rates of replication complex displacement during replication of the complementary sense gene encoding genome regions [55]. If multiple similar but non-identical geminivirus genomes are present within the same nucleus then re-initiation of replication from partially replicated virion strands on a new template molecule (occurring via the recombination dependent pathway of geminivirus replication [53]) could result in an increased prevalence of detectable recombination events across the C-sense ORFs and the IR.

Surprisingly, the recombination cold-spots within the IR and at the V1/C3 interface are at sites that are clearly detectable as recombination hot-spots within studies of natural monopartite begomoviruses ([8]; compare the blue shaded regions of the breakpoint distribution plot with the pink bars above the plot in Figure 3). These differences may reflect the fact that our experiments involve only a single pair of parents within a single host whereas the distribution of breakpoints seen in field isolated viruses is attributable to hundreds of parent pairs recombining in hundreds of different host species. If selection influenced both the experimental and natural recombination patterns to a similar degree, the differences between the patterns would indicate that for our particular experimental host-virus combination, recombination breakpoints falling at the V1/C3 interface and at the IR cold-spot are either (1) unusually deleterious relative to analogous events between other begomovirus pairs in different host species or (2) simply occur at a mechanistically lower frequency than is generally the case with other virus-pair and host combinations.

It is obvious that the viruses in our experiment experienced selective processes that were probably substantially different to those of natural viruses which, besides infecting a variety of different host types, must generally also persistently maintain a high degree of transmission fitness. Nevertheless, the fact that the recombination patterns detected in our experiment largely match those observed for natural recombinants implies that, despite its simplicity, the experiment clearly recaptures many of the mechanistic and selective processes shaping patterns of recombination seen in natural viruses.

### Mechanistic processes influencing recombination patterns: Local degrees of sequence similarity

Before considering the influence of natural selection on the recombination patterns that we observed, we sought to identify mechanistic factors that might have contributed to variations in recombination frequencies observed across the TYX and TOX genomes. Two factors that have been frequently associated with variations in basal recombination rates across viral genomes are local degrees of sequence similarity [1], [2], [4], [56]–[58] and the presence of nucleic acid secondary structures [5], [26]. Whereas degrees of similarity shared by potentially recombining sequences will obviously impact the efficiency of homologous recombination [1], [2], [4], [56]–[58], secondary structures may stall replication and increase the probability of recombination breakpoints falling within structured genome regions [5], [26].

**Figure 4:**
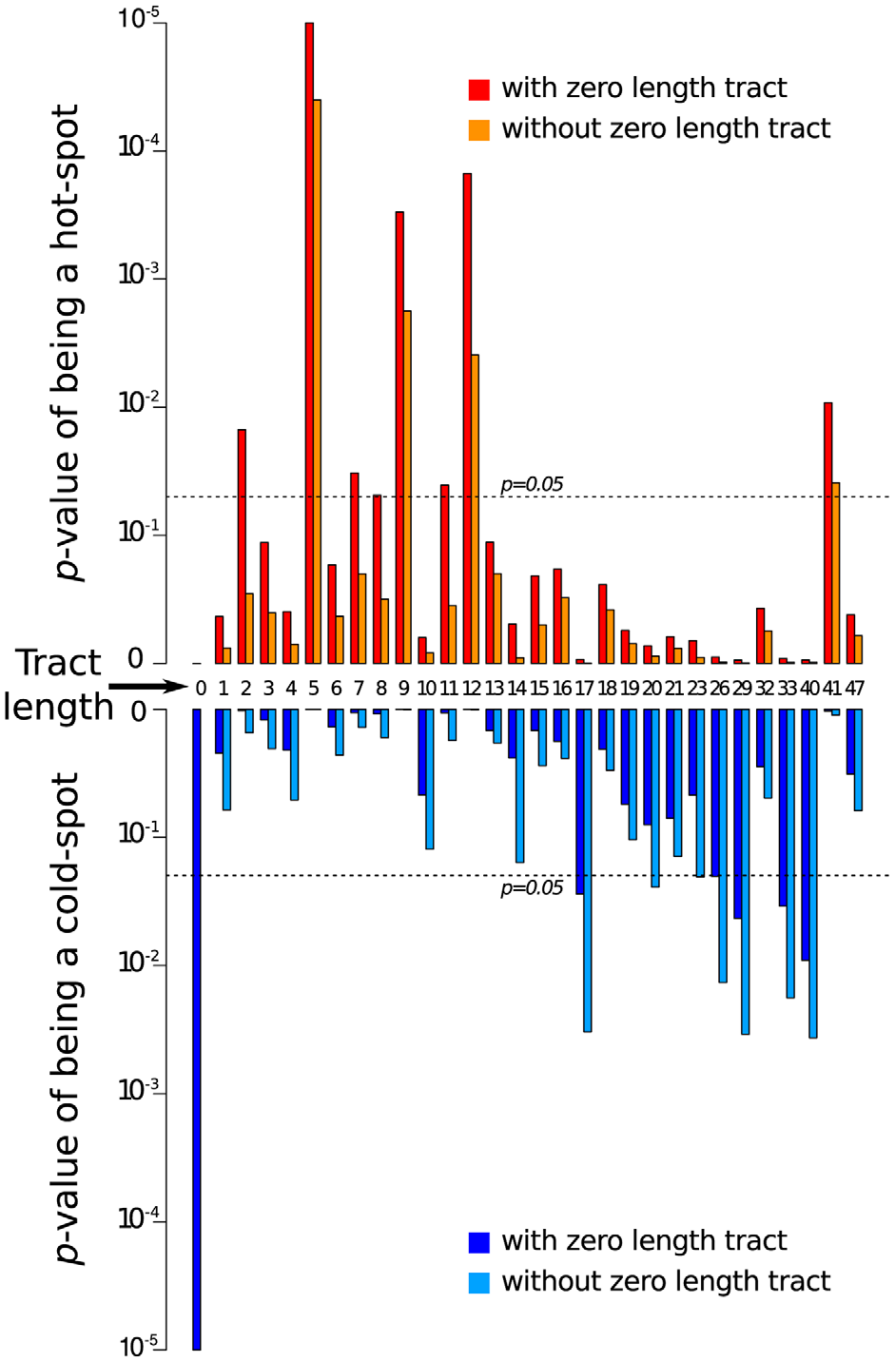
Breakpoints arising during mixed infections tend to fall significantly more frequently at sites where *Tomato yellow leaf curl virus* (TYX) and *Tomato leaf curl Comoros virus* (TOX) share identical stretches of between 5 and 12 nucleotides and significantly less frequently at sites where they share 17 or more identical nucleotides. The high frequency of recombination breakpoints within the 41 nucleotide long site category is attributable to this being the virion strand origin of replication (a known recombination hotspot).

We therefore tested the influence of the length of identical nucleotide tracts between the parental viruses on the numbers of breakpoints falling within those tracts and detected four different tract-size classes: under conditions of completely random recombination, (1) zero length tract sizes (i.e. when breakpoints occur between two sites that are polymorphic) are statistically far less prone to recombination than can be accounted for by chance (*p*<0.00001, Figure 4); (2) intermediate tracts of perfect identity between 2 and 12 nucleotides long tended to be more recombinogenic than expected (there are seven *p*-values <0.05 for tract lengths in this size class when zero length tract sizes are considered and three when they are excluded); (3) large tracts of perfect identity greater than 16 nucleotides long tend to be less recombinogenic than expected (five *p*-values for tract lengths in this size class < 0.05 when zero length tract sizes are considered and six when they are excluded) and; (4) the 41 nucleotide tract of 100% TYX and TOX identity surrounding the stem-loop region encounters more recombination breakpoints than can be accounted for by chance (*p* = 0.0092 when zero length tracts are included in the analysis and *p* = 0. 0388 when they are excluded; Figure 4). The recombination hot-spot at the virion strand replication origin and the cold-spots at zero length tracts were entirely expected given that recombination in geminiviruses is believed to occur by a strongly homology dependent double stranded DNA break repair mechanism during which monomeric genomes are replicationally released from multimeric genome concatemers [55]. Although a similar tendency for decreasing recombination frequencies in fragments over a certain length having been reported in retroviruses (which recombine by a replicational copy-choice mechanism; [56]), we were unable to find any plausible explanation as to why recombination in begomoviruses should occur more frequently at 5–12 nucleotides long identical tracts than it is does at identical tracts >16 nucleotides long.

### Mechanistic processes influencing recombination patterns: Genomic secondary structures

Mounting evidence indicates that recombination breakpoints observable in many single stranded viral genomes tend to co-localise with base-paired nucleotides within genomic secondary structures [2], [5], [26]. We therefore tested whether in our experiment recombination breakpoints fell more frequently than could be accounted for by chance at sites predicted to be base-paired within the thermodynamically most favourable secondary structures of the ssDNA TYX and TOX genomes. Applying the same test used in Simon-Loriere *et al*. [7] to detect a clear association between breakpoint sites and secondary structures within HIV genomes, we analysed breakpoint distributions in relation to the distributions of TYX and TOX genomic sites inferred using the program UNAFold to be involved in base-pairing within genomic secondary structures [40]. Regardless of the subsets of sites that we examined (see M&M and Table S2) we detected no clear tendency for recombination breakpoints to fall at sites associated with predicted ssDNA genomic secondary structures. Although we observed a marginally significant tendency for breakpoints to cluster at sites where TOX had a strongly predicted secondary structure but TYX did not (*p* = 0.0579; Table S2), it is important to stress that we performed three separate tests and that we would have expected an approximately one in six chance of finding at least one *p*-value this close to significance even in the absence of any real associations.

It is perhaps not surprising that we found no strong correlation between the positions of recombination breakpoints and secondary structures in that much (if not most) begomovirus recombination is probably a by-product of double stranded break repair pathways [55]. Clear associations seen between secondary structures and recombination breakpoints positions observed in some RNA viruses [2], [5] have been attributed to copy-choice recombination mechanisms where secondary structures cause either reverse transcriptase or RNA dependent polymerase complexes to dissociate from template molecules. If these stalled complexes reengage a template other than those on which they had originally been situated then the synthesised RNA or DNA strands will be recombinant. Although the possibility remains that in begomoviruses copy-choice type recombination occurs due to secondary structure induced stalling of DNA replication complexes, the breakpoint distributions attributable to this mechanism would be largely obscured by those attributable to double stranded break repair mechanisms.

### The influence of selection on detectable breakpoint distributions

Besides the potential influences of mechanistic factors such as local degrees of sequence similarity and nucleic acid secondary structure on the recombination breakpoint distributions observed in our experiment, we were also interested in determining whether there existed evidence of this distribution having been influenced by natural selection. As with all life on Earth co-evolved sequence specific DNA-DNA, protein-protein and protein-DNA interactions have a central role in the molecular biology of begomoviruses and it is reasonable to suspect that natural selection will strongly disfavour the survival of recombinant begomovirus genomes in which these interactions are disrupted. For example, while intra-protein amino acid interactions determine how proteins fold, inter-protein amino acid interactions determine how proteins oligomerise or form complexes. Importantly, recombination can potentially disrupt such co-evolved interactions by bringing together pairs of amino acids that do not interact properly during protein folding, oligomerisation and complex formation [9].

We therefore tested for evidence of observed recombination events that occurred during our experiments being generally less disruptive of co-evolved intra-genome interaction networks than could be accounted for by chance. Specifically the interactions that we considered included (1) amino acid interactions within the 118 N-terminal amino acids of Rep and within the 196 amino acids of CP, (2) nucleotide interactions within predicted genomic ssDNA structures and (3) known protein-protein and protein DNA interactions between different genome regions.

### The influence of selection on recombination patterns: Protein folding disruption

Previous analyses of begomoviruses [8], other ssDNA viruses [41] and HIV [5], [7], have indicated that, assuming random recombination, natural recombinants tend to display lower degrees of predicted recombination induced protein folding disruption than can be accounted for by chance. Whereas recombination breakpoints in these viruses display a marked tendency to fall outside of coding regions, when they do fall within genes they tend to occur either on the edges of genes [5], [41], or near domain boundaries where they have a minimal impact on protein folding [7], [8].

Using the breakpoint clustering tests of Lefeuvre *et al*. [41], we detected a clear tendency for recombination breakpoints to preferentially fall outside of coding regions (*p* = 0.0001 and *p* = 0.1174 for the 50 sequence FULL and the 18 sequence FIT datasets respectively). It was also clearly evident that recombination breakpoints that did fall within genes tended to fall within the 5′ 12.5% and 3′ 12.5% of nucleotides of the genes significantly more frequently than they did within the rest of the genes (*p*<0.0001 and *p* = 0.0061 for the FULL and FIT datasets respectively). Although differences in the *p*-values obtained with the FULL and FIT datasets most likely reflect the greater sample size in the FULL dataset, it is possible that they indicate that selection has not been entirely responsible for these observed breakpoint patterns. For example, the recombination breakpoint hotspot at the virion strand replication origin automatically predisposes the IR to have more recombination breakpoints than the remainder of the genome.

We therefore focused on the C1 and V1 genes for which corresponding atomic resolution 3D protein structure models are available to allow us to more directly test for evidence of selection acting against recombinants expressing misfolded proteins. Specifically, we tested whether recombinants observed in our experiment tended to express chimaeric Rep and CP molecules with less potential folding disruption than what would have been expected if recombination breakpoints within these coding regions occurred at random. Considering all 50 unique recombination patterns within the FULL dataset we detected no evidence of the recombinants expressing Rep and CP molecules with better preservation of intra-protein amino acid interactions than would have been expected if recombination breakpoints occurred at random (*p* = 0.13 and 0.89 for Rep and CP respectively). While this result implied that selection against protein folding disruption had not obviously influenced the over-all distribution of observed recombination breakpoints falling within the C1 and V1 ORFS, it was anticipated that low frequency, potentially transient/defective recombinant forms that we included amongst the 50 FULL dataset recombinants may have obscured this signal of natural selection.

When we repeated the analysis with only the FIT dataset containing 18 recombinants that were viable enough to have been sampled multiple times during the experiment we detected a clear signal indicating that the recombination breakpoint patterns within their C1 ORFs were less disruptive of protein folding than expected by chance (*p* = 0.016). Although we detected no such signal within the V1 ORF (*p* = 0.47), this negative result was expected in that (1) TYX and TOX express CPs that differ at only two potentially interacting amino acid sites (implying that even the most disruptive recombinant would only result in two potential interacting residue perturbations); and (2) among the FIT recombinants there were only 8 detected recombination breakpoints within V1.

It is nevertheless interesting that we detected significant avoidance of Rep folding disruption amongst what we assume to be the fittest subset of recombinants and no such signal amongst the total set of recombinants. This implies that the selective processes acting against recombinants expressing improperly folded chimaeric proteins that are detectable in global begomovirus populations, are clearly operational during the short-term evolution of recombinants within the hosts where they originate.

### The influence of selection on recombination patterns: ssDNA folding disruption

The stability and distribution of DNA and RNA secondary structures are important fitness determinants in many viral species [59]–[62], including geminiviruses [63], [64]. Since nucleotide interactions within evolutionarily conserved secondary structures within the TYX and TOX genomes are another potential subset of co-evolved intra-genome interactions that might be disrupted by recombination, we investigated whether the recombinant genomes that arose during our experiment displayed evidence of selection favouring the preservation of base-pairing interactions within genomic secondary structures.

We first investigated whether the over-all stability of secondary structures within the 50 unique recombinant genomes observed in our experiment were significantly different from what would have been expected given random recombination. Specifically we compared the predicted MFE of the real recombinants to sets of simulated recombinants displaying both similar spacing between recombination breakpoints and the same numbers of TYX and TOX derived nucleotides as the real recombinants. Although we found some variation in the estimated MFEs of recombinants and parental viruses (i.e. recombination was predicted to have had some influence on MFE estimates), there were no significant differences in MFE estimates between the simulated and real recombinants (for either the total 50 sequence FULL set or the 18 sequence FIT set). This indicated that the degree to which overall secondary structural stability (as measured by genomic MFEs) is conserved between parental and recombinant sequences is not an overwhelmingly significant factor determining the short-term survival of recombinants in the hosts where they originate.

This result did not imply, however, that there was no evidence of selection favouring the maintenance among recombinants of specific nucleotide interactions (such as those within the predicted hairpin structure at the origin of virion strand replication). To test for such evidence we adopted exactly the same approach used in the SCHEMA-based protein folding disruption tests but applied it to predicted nucleotide-nucleotide contact maps inferred from predicted MFE secondary structures instead of amino acid-amino acid contact maps inferred from protein 3D structure models. Also, unlike the protein folding disruption tests we inferred separate secondary structures both for the parental TYX and TOX genomes and for the real and simulated recombinants. This latter point meant that we were able to differentiate between two distinct types of ssDNA folding disruption: (1) disruptions where the recombinants have potentially aberrant nucleotide base pairings that are not found in either the TYX or TOX secondary structures and (2) disruptions where recombinants are missing base pairings that are found within the TYX and TOX secondary structures.

Both of these SCHEMA based ssDNA folding disruption tests indicated clear evidence of the recombinants in our experiment displaying lower degrees of ssDNA folding disruption than was expected by chance (*p* = 4.9×10^−3^ for the FULL dataset and *p* = 3.7×10^−2^ for the FIT dataset with test (1) and *p* = 2.4×10^−5^ for the FULL dataset and *p* = 7.8×10^−5^ for the FIT dataset with test (2). It is very noteworthy, however, that by far the strongest signal of secondary structure preservation came from the test looking at the preservation of parent-like base-pairing patterns. While it is not acceptable to equate these estimated *p*-values with the strength of the selective process that have probably generated these signals, the vastly different strengths of the signals in tests 1 and 2 nevertheless suggests that selection might not disfavour the survival of recombinants with novel non-parental base pairings nearly as strongly as it disfavours those with broken parental-base pairings.

The simple fact that we have detected evidence that observed recombinants tend to have lower degrees of ssDNA folding disruption that might be expected by chance also implies that in begomoviruses (but possibly also in other ssDNA viruses too) many of the genomic secondary structures that are readily predictable using currently available DNA folding methods are probably evolutionarily relevant. Besides the probable hairpin structure at the virion strand origin of replication of *Tomato golden mosaic virus* [63] and one other structure within the *rep* gene of *Maize streak virus* [64], there remains no direct experimental evidence for the existence of widespread and biologically relevant base-pairing within single stranded geminivirus genomes. Our results, however, strongly suggest that if such evidence is sought it will probably be found.

### The influence of selection of recombination patterns: Conservation of long-range interactions

While our previous analyses attempted to determine the influences of short range interactions on recombination patterns (such as those between amino acids within folded proteins and nucleotides within DNA secondary structures), we realised that the patterns of recombination that emerged within our experiment were potentially also influenced by longer-range inter-protein or protein-DNA interactions. We therefore devised a permutation-based genetic association test to both determine whether there existed any evidence that these known long-range interactions were preferentially preserved within the observed recombinants, and indicate whether these recombinants could be used to reveal other currently unknown intra-genome interactions.

If we assume that intra-genome interactions have co-evolved over time and that, if the partners of a co-evolved interacting pair (such as two encoded amino acids interacting within a folded protein or an encoded protein motif and the DNA sequence it recognizes) are separated by a recombination event, we would expect the recombination event to incur a fitness cost if the transferred half of the interacting pair did not work as well with its new interacting partner as it did with its old co-evolved one. We would therefore expect that recombinants in which different halves of interacting pairs have been derived from different parental sequences, would be selected against and would occur at lower than expected frequencies in the population.

**Figure 5:**
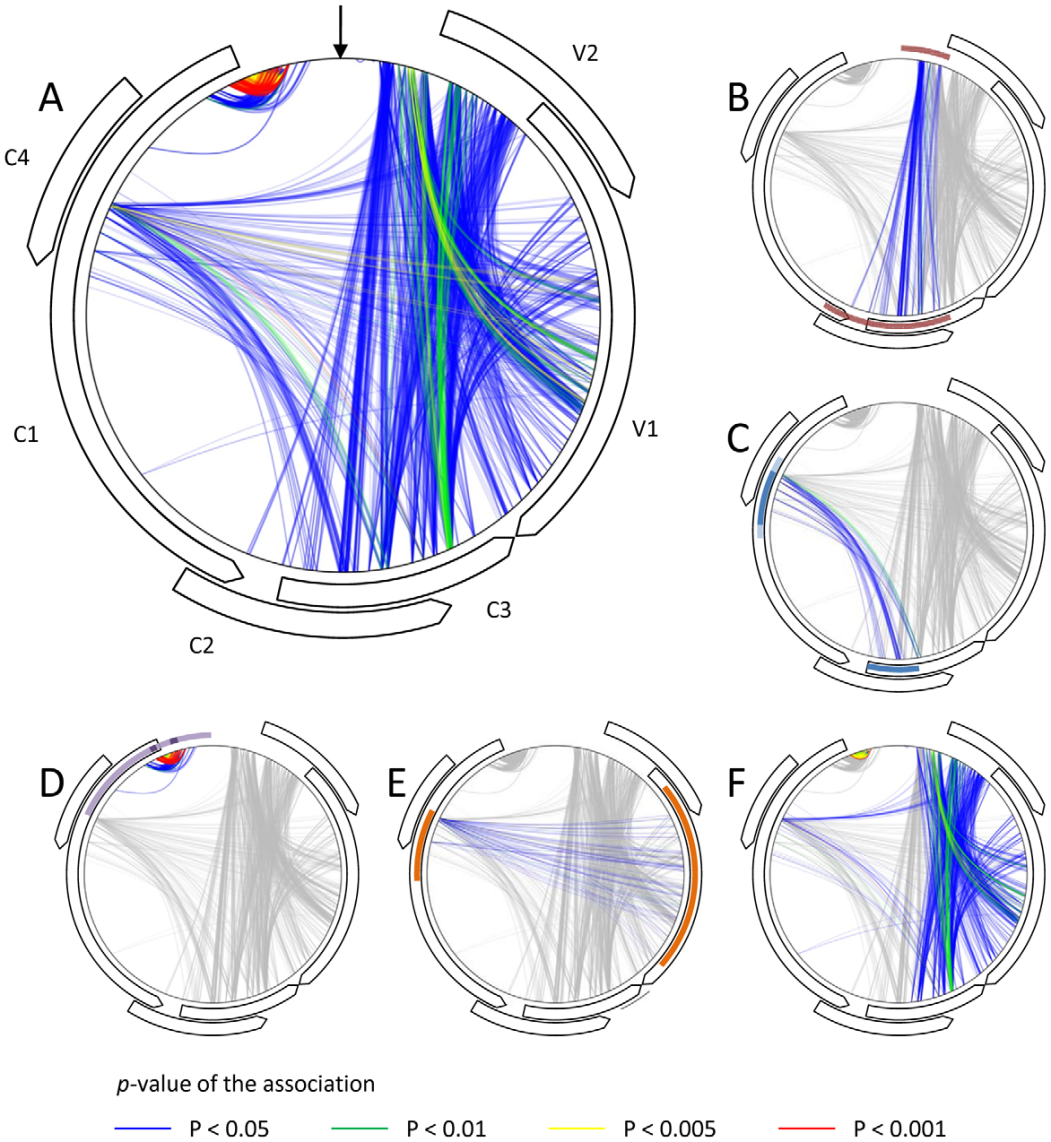
Schematic representation of nucleotide associations implied by non-random patterns of recombination that occur during *Tomato yellow leaf curl virus* (TYX) and *Tomato leaf curl Comoros virus* (TOX) co-infections. Curved arrows indicate the positions of open reading frames (ORFs) and the vertical black arrow indicates the position of the origin of virion strand replication. The indicated *p*-values (represented by different colours) are for a pair permutation-based association tests such that, for example, a red line between two genomic sites indicates that a *p*-value <0.001 was obtained for both the Chi-square based association test, and the test for lower than expected mixtures of TOX and TYX derived polymorphisms at the indicated pair of sites (i.e. both members of the pair tend to be inherited from the same parental virus). (**A**) All of the detected associations. (**B**) Associations potentially caused by the known interaction between the C3 gene product (the transcription activator protein) and the V1 and V2 gene promoter (the currently mapped sites for this interaction and the others below are indicated by coloured arcs; [32]). (**C**) The known interaction between the C1 gene product (the replication associated protein) and the C3 gene product (the replication enhancer protein; [30]) (**D**) The known interaction between the C1 gene product and the C1 proximal portion of the intergenic region (indicated by light purple arcs; [33]–[34]). The bioinformatically mapped sites of so-called iterons (within the intergenic region) and iteron related domains (within the replication associated protein) believed to be the specificity determinants of this interaction are indicated by darker purple arcs [31]. (**E**) The known interaction between the V1 gene product (the coat protein) and the C1 gene product [50]. (**F**) Associations potentially caused by as yet unknown interactions between the V1 and V2 genes/gene products, the V1 gene/gene product and the intergenic region, and between the C3 and V2 genes/gene products.

We therefore sought to identify site pairs at which nucleotides tended to be co-inherited from the same parent more frequently than could be accounted for by chance such that mixtures of TYX and TOX derived polymorphisms were significantly under-represented at these sites in our recombinant population. The sites where these “mixed” polymorphism pairs were under-represented were compiled on an association map (Figure 5). Collectively we detected 4759 site-pairs (3.9% of the total site pairs queried) at which mixed TYX and TOX polymorphism combinations were significantly under-represented (i.e. with an associated permutation *p*-value < 0.05 for both of the association tests that we performed).

Most of these site pairs included one polymorphic site within the IR and the other in either (1) the overlapping region of the C2/C3 ORFs, (2) the overlapping region of the C4 and C1 ORFs, (3) the 5′ region of C1 and (4) the V1 ORF (Figure 5A). It is important to note here that detecting 3.9% of site-pairs with fewer than expected mixed TYX-TOX polymorphisms is in fact within the range expected by chance alone given a *p*-value cutoff of 0.05. It should be remembered, however, that these site-pairs were selected based on two separate tests both with a 0.05 *p*-value cutoff and that the anticipated rate of falsely inferred interactions is therefore expected to be substantially lower than 5%. Since we do not know what the expected false positive rate is with using the consensus of these two tests we suggest when consulting Figure 5 that only those site pairs identified with a p-value cutoff of 0.005 for both tests (orange and yellow lines) should be individually interpreted as displaying strong evidence of interacting with one another.

If the under-representation of particular “mixed” polymorphism pairs indicates that the genome regions carrying these pairs are probably involved in co-evolved intra-genome interactions, then at least some of the regions known to interact within geminivirus genomes should be among these site-pairs. This is in fact exactly what we find. Namely, (1) the inferred IR/C2/C3 interactions inferred from our recombination analysis match the TrAP-V1 promoter interaction sites recently mapped by Lacatus & Sunter [32] (Figure 5B), (2) the inferred C2/C3- C4/C1 interactions match with previously mapped REn and Rep interaction sites ([30]; Figure 5C), (3) the inferred 5′C1 - IR interaction correspond precisely to the Rep-iteron-related domain-iteron interaction sites proposed by Argüello-Astorga & Ruiz-Medrano [31] (Figure 5D) and (4) inferred V1-C1 interactions correspond with the Rep-CP interaction sites mapped by Malik et al. [50] (Figure 5E).

This preferential co-inheritance from a single parent of genome site pairs that are known to interact with one another clearly supports our hypothesis that, within our experiment, natural selection has probably disfavoured the survival of recombinants in which long-range intra-genome interactions have been disrupted.

Perhaps more importantly, however, the recovery by our association tests of these known interactions provides compelling evidence that applying these tests to recombination experiments such as we have performed can potentially also uncover unknown intra-genome interactions such as those indicated in Figure 5F.

When considering evidence provided by our association test for such unknown interactions it is important to stress that, as with most genetic association tests, ours suffers from an inescapable degree of genetic hitch-hiking that is expected to result in polymorphic sites which are physically closely linked on the genome displaying the same or very similar association patterns with sites in the remainder of the genome. This is because genome fragments that are exchanged by recombination usually carry multiple polymorphisms. This has two important consequences for our test. The first is that whereas the test might be capable of detecting long-range interactions, it is expected to have far less power to detect interactions between sites that are situated close to one another on the genome. The second is that many apparent interactions revealed by the test could be indirect. For example, the test might indicate that a pair of sites, A and B are always co-inherited from the same parent and are therefore probably interacting, but what is actually happening is that sites A and B are both in independently interacting with a third site, C. Importantly, however, as the number of analysed recombination events increases and the degree of genetic hitch-hiking decreases, one would expect both increases in the over-all accuracy with which interactions can be mapped by this approach, and increases in the power with which shorter-range interactions can be detected: Perhaps even including short range biologically relevant intra-protein amino acid contacts or structurally important nucleotide interactions.

### Concluding remarks

Our recombination experiments yielded an array of recombinant genomes that far exceeded in their sheer numbers and complexity those encountered in other previously described geminiviral evolution experiments. Although polymorphisms derived from one or the other of the parental genomes were clearly selectively favoured at ∼64% of polymorphic sites within the arising recombinants, no highly deterministic single “recombinant solution” emerged from our experiment. Whereas a single prevalent recombinant solution such as has been found in other geminivirus recombination experiments [26], [51], [52] would have indicated the existence of a fitness landscape dominated by a single sharp peak, the unparalleled diversity of recombinants observed in our experiment possibly reflects the relative “flatness” of the tomato fitness-landscape over which the TYX and TOX recombinants evolved during our experiment. Although we cannot know whether over the long-term upon this flattish landscape some of the recombinants we observed could have out-competed their parental genomes under natural conditions, it is apparent that TYX and TOX are possibly more “genetically compatible” when it comes to making recombinants than other geminivirus combinations that have been tested in the past [13], [26], [65].

We show here that within begomovirus co-infections, local degrees of sequence similarity, but not genomic secondary structure, strongly influences the genesis of recombinant sequences. Once recombinants are produced, however, we additionally show that by determining which of these survive, natural selection profoundly influences the over-all patterns of recombination that emerge. While selection apparently favours the survival of recombinants with particular combinations of host adaptive polymorphisms, we show that it also clearly favours the survival of recombinant genomes within which various categories of intra-genome interaction are preserved. Relative to simulated recombinants, those emerging during our experiment tended to display (1) better preservation of amino acid interactions within their folded proteins, (2) better preservation of nucleotide interactions within predicted genomic secondary structures, and (3) better preservation of known long-range intra-genome protein-protein and protein-DNA interactions. We in fact show that the imprint left by natural selection on the patterns of recombination that emerged in these experiments was so profound that the patterns could be used to trace key features of the interaction networks encoded within the two parental genomes.

Finally, there is no reason that such recombination based approaches to high throughput mapping of either host adaptive polymorphisms or genomic interactions could not be applied to any recombinogenic virus species (for example most double stranded DNA viruses, retroviruses or positive strand RNA viruses). In cases where viruses do not naturally recombine (such as with many negative strand RNA viruses), random recombinants could be synthesised *en masse* and used to co-infect either host cells in culture or whole organisms. Besides illuminating the internal workings of viral genomes, systematic screening of different virus and host combinations would reveal the genetically compatible virus pair and host species combinations that are likely to produce the emergent recombinants of the future.

## Supporting Information

Figure S1 Schematic representation of the methodology used to detect associations between polymorphic nucleotide sites within the TYX and TOX derived recombinant genomes associations in the recombinant alignment.
(TIF)

Figure S2 The distribution of recombination breakpoints detected within the 114 recombinant sequences emerging during *Tomato yellow leaf curl virus* (TYX) and *Tomato leaf curl Comoros virus* (TOX) co-infections. All detectable unique breakpoint positions are indicated by small vertical lines at the top of the graph. A 50 (panel A), 75 (panel B), 100 (panel C) and 200 (panel D) nucleotide window was moved along the alignment one nucleotide at a time and the number of breakpoints detected within the window region was counted and plotted (solid line). The upper and lower broken lines respectively indicate 99% and 95% confidence thresholds for globally significant breakpoint clusters. Light and dark grey areas respectively indicate the expected 99 and 95 percentiles of expected recombination breakpoint clustering assuming random recombination. Whereas red areas indicate recombination hot-spots, blue areas represent recombination cold-spots. The positions of open reading frames (ORFs; horizontal arrows) and intergenic regions (IR) are represented on the top of the graph. Pink and blue horizontal lines beneath the ORF map respectively delineate the positions of recombination hot and cold spots detectable within begomovirus genomes sampled from nature (from Lefeuvre et al. [8]).
(TIF)

Protocol S1 Supplementary methods and materials.
(DOC)

Table S1 Summary of full genome sequences examined in this study.
(DOC)

Table S2 Testing for an association between predicted genomic secondary structures and recombination breakpoint positions.
(DOC)
