# Supplementary figures and images for: Complex Recombination Patterns Arising during Geminivirus Coinfections Preserve and Demarcate Biologically Important Intra-Genome Interaction Networks
Source: PLoS Pathog. 2011 Sep 15;7(9):e1002203. doi: 10.1371/journal.ppat.1002203 (PMC3174254; doi:10.1371/journal.ppat.1002203)

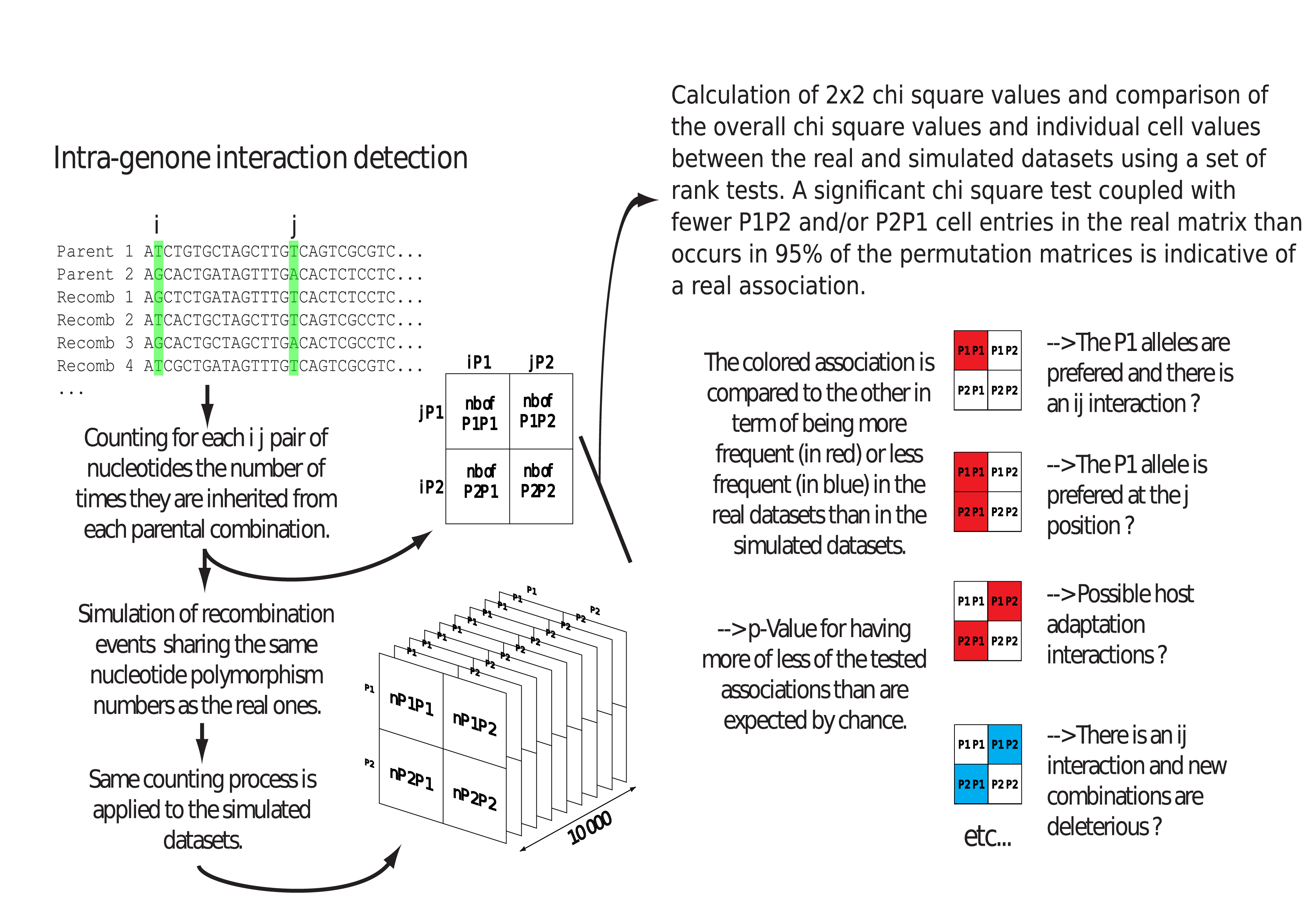

Supplement: Figure S1 — Schematic representation of the methodology used to detect associations between polymorphic nucleotide sites within the TYX and TOX derived recombinant genomes associations in the recombinant alignment. (TIF) [file ppat.1002203.s001.tif]

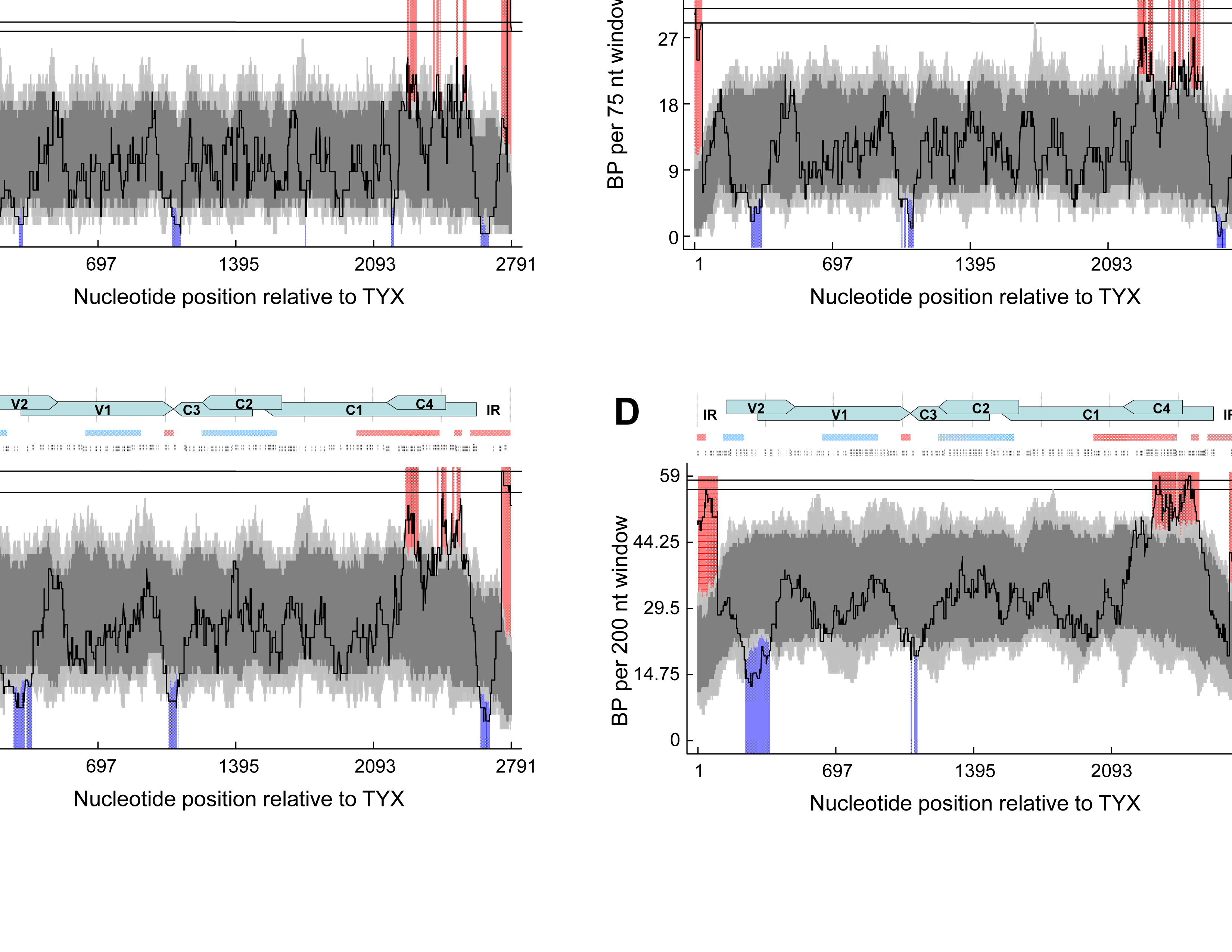

Supplement: Figure S2 — The distribution of recombination breakpoints detected within the 114 recombinant sequences emerging during Tomato yellow leaf curl virus (TYX) and Tomato leaf curl Comoros virus (TOX) co-infections. All detectable unique breakpoint positions are indicated by small vertical lines at the top of the graph. A 50 (panel A), 75 (panel B), 100 (panel C) and 200 (panel D) nucleotide window was moved along the alignment one nucleotide at a time and the number of breakpoints detected within the window region was counted and plotted (solid line). The upper and lower broken lines respectively indicate 99% and 95% confidence thresholds for globally significant breakpoint clusters. Light and dark grey areas respectively indicate the expected 99 and 95 percentiles of expected recombination breakpoint clustering assuming random recombination. Whereas red areas indicate recombination hot-spots, blue areas represent recombination cold-spots. The positions of open reading frames (ORFs; horizontal arrows) and intergenic regions (IR) are represented on the top of the graph. Pink and blue horizontal lines beneath the ORF map respectively delineate the positions of recombination hot and cold spots detectable within begomovirus genomes sampled from nature (from Lefeuvre et al. [8]). (TIF) [file ppat.1002203.s002.tif]
