# Supplementary material for: Complex Recombination Patterns Arising during Geminivirus Coinfections Preserve and Demarcate Biologically Important Intra-Genome Interaction Networks
Source: PLoS Pathog. 2011 Sep 15;7(9):e1002203. doi: 10.1371/journal.ppat.1002203 (PMC3174254; doi:10.1371/journal.ppat.1002203)
Supplement: Protocol S1 — Supplementary methods and materials. (DOC) [file ppat.1002203.s003.doc]

**Protocol S1**

**Complex recombination patterns arising during geminivirus coinfections preserve and demarcate biologically important intra-genome interaction networks**

D.P. Martin^a^ , P. Lefeuvre^a,b^, A. Varsani^,c,d,e^, M. Hoareau^b^, J.Y. Semegni^a^, B. Dijoux^b^, C. Vincent^b^, B. Reynaud^b^ and J.-M. Lett^b^

^a^Institute of Infectious Diseases and Molecular Medicine, University of Cape Town, Observatory 7925, South Africa

^b^CIRAD, UMR 53 PVBMT CIRAD-Université de la Réunion, Pôle de Protection des Plantes, Ligne Paradis, 97410, Saint Pierre, La Réunion, France

^c^School of Biological Sciences, University of Canterbury, Private Bag 4800, Christchurch, 8140, New Zealand.

^d^Biomolecular Interaction Centre, University of Canterbury, Private Bag 4800, Christchurch, 8140, New Zealand.

^e^Electron Microscope Unit, University of Cape Town, Rondebosch, Cape Town.7701, South Africa

**Detection of recombination breakpoint hot- and cold-spots**

We have previously devised a permutation test to identify recombination hot and cold-spots within viral genomes. This permutation test takes into account the observed distribution of polymorphic sites between the two parental viruses (TYX and TOX in this case) and randomly maps a set of detected recombination events to this distribution such that each real event has a corresponding randomly mapped event involving a transferred sequence tract containing an identical number of polymorphic sites. In doing so, the test accounts for the fact that local variations in degrees of sequence similarity influenced the accuracy with which recombination breakpoints could be mapped. In the current study after determining the actual recombination breakpoint distribution from our experimental recombinants, this random breakpoint placement process was repeated 10 000 times. The actual distribution of breakpoints was compared to that of the 10 000 permuted distributions using two tests in which: (1) Globally significant breakpoint clusters were identified as genome regions that contained a higher breakpoint densities than could be found at *any genome region* in more than 95% of the 10 000 permuted breakpoint density plots; (2) Locally significant breakpoint clusters were identified as genome regions that contained higher recombination breakpoint densities than could be found in the *same genome regions* of 99% of the 10 000 permuted recombination breakpoint density plots.

**Determining the influence of local degrees of sequence similarity on recombination breakpoint frequencies**

In various studies involving other viruses it has been demonstrated that, during homologous dependent recombination, the frequency of recombination breakpoints occurring at a specific genomic site is strongly influence by the number of identical nucleotides shared between the parental sequences at that site [1-5]. We split the pairwise TYX-TOX sequence alignment up into a set of site categories based on the numbers of contiguous nucleotides that were identical between the two sequences. For example, recombination breakpoint sites within a run of 10 contiguous nucleotides that were identical between TYX and TOX were placed within site category 10 whereas those that fell between two adjacent non-identical nucleotides were placed within site category 0. We used a permutation test to determine whether the observed frequency of recombination breakpoints at each of these site categories was significantly different from those expected at these sites assuming that recombination breakpoints were randomly distributed and uninfluenced by local degrees of sequence similarity. The closest that each recombination breakpoint detected in our experiment could be mapped to the actual site where it occurred was to the two sites on either side of the breakpoint where TYX and TOX were polymorphic. For each of the ten plants examined in our experiment we mapped breakpoints to runs of sites at which TYX and TOX were identical. In cases where multiple breakpoints were mapped to the same run of sites in an individual plant, only a single breakpoint was counted as having occurred at the sites. This was to ensure that when counting how many breakpoints occurred in the viruses isolated from each plant we counted each actual breakpoint only once irrespective of how many of the sampled genomes it occurred within. Given the numbers of unique breakpoints detected in each plant and the lengths of the TYX-TOX identical genome fragments where these were detected, we randomly shuffled the breakpoint positions in 10^6^ permuted datasets. As in the real dataset these permuted datasets each contained ten sets of mapped breakpoints each of which corresponded to one of the ten plants in the experiment. The number of breakpoints randomly distributed in each of the ten sets was the same as that in the corresponding set in the actual experiment and, as with the real dataset, in each of the ten sets only one breakpoint was permitted per genomic site.

For example, testing whether in our experiment breakpoints occurred more frequently than could be accounted for by chance at sites where TYX and TOX share runs of five identical sites simply involved: (1) Counting the number of times breakpoints were mapped to such sites in the real dataset; (2) making the same count for each of the 10^6^ permuted datasets; (3) counting the number of times the permuted datasets had counts higher than or equal to that of the real dataset; and (4) dividing by the number of permutations. The proportion thus obtained is an estimate of the probability that breakpoints do not occur preferentially at sites where TYX and TOX share five identical nucleotides.

**Determining the influence of predicted local ssDNA structure on recombination breakpoint frequencies**

Evidence is mounting that recombination breakpoint patterns observable in many single stranded viral genomes are influenced by nucleotide base pairing within their thermodynamically most favourable folded structures [1,6,7]. A permutation test of recombination breakpoint clustering based on those described in [6] and [8], was used to determine associations between RNA secondary structure and breakpoint clustering. These tests are themselves a modification of the breakpoint clustering test of [9] that is described above and accounted for uncertainties in recombination breakpoint site identification due to the underlying degree of sequence conservation (recombination breakpoints can be more accurately mapped in more divergent regions). These tests can be used to indicate whether recombination breakpoints are significantly more or less clustered within specified pairs of genome regions. For example, in the present study they were used to compare “structured” genome sites (i.e. those that are predicted to be base paired within the folded secondary structure of one or both parental genomes) with “unstructured” genome sites (i.e. those that are predicted not to be base paired within either of the parental genomes). In all cases, observed breakpoint distributions within the two site categories were compared with breakpoint distributions determined for 10 000 simulated datasets each displaying precisely the same number and character of recombination signals (spacing between breakpoint positions, degrees of parental sequence relatedness, and numbers of sequences carrying evidence of recombination), but with breakpoint positions randomized (see above for the recombination hot- and cold-spot test). When comparing, for example, breakpoint densities within structured region A with those in unstructured region B, the sum of breakpoint numbers observed within regions A and B in the real dataset were randomly distributed between the regions in each of the 10 000 simulated datasets. Simulated datasets in which the number of breakpoints in region A was equal to or greater than the number observed in region A of the real dataset were counted. This count was then divided by 1000 to yield the probability that breakpoints were not significantly more clustered in region A in the real dataset than could be accounted for by chance. The inverse test (i.e. testing to determine whether breakpoints were significantly less clustered in region B than could be accounted for by chance), was also carried out.

**Determining the influence of genome location on recombination breakpoint frequencies**

This was carried out using the same breakpoint clustering tests as those mentioned directly above except that instead of structured and non-structured genome regions being compared, the comparisons were coding vs non-coding regions and the ending 25% of genes vs. the middle 75% of genes.

**SCHEMA-based protein folding disruption tests (after Lefeuvre et al., 2007)**

SCHEMA [10] is a method designed to infer the approximate degree of protein folding disruption expected within a chimaeric protein. It takes a PDB protein structure file (in this case corresponding to the CP and the Rep N-terminus) and the parental amino acid sequences (in this case those of TYX and TOX) as inputs. It uses the protein structural information to fold the parental amino acid sequences using homology modelling and then identifies potentially interacting amino acid pairs based on their proximity (in this case within 4.5 Å) within the resulting folds. The resulting amino acid contact map can be used to determine the degree of fold disruption expected in any conceivable chimaera of the parental amino acid sequences. For all the amino acid residues that are potentially interacting within a folded chimaeric protein, all that needs be done is to count the number of instances where the interacting pairs are non-parental. Non-parental interacting amino acid pairs arise when the parental molecules differ from one another at two potentially interacting amino acid residues and the chimaera inherits one-half of the potentially interacting pair from one parent and the other half from the other parent. Counts of these non-interacting pairs in chimaeric proteins, called “E” values, have been shown to correlate directly with degrees of fold disruption experienced by the proteins [10]. The value of E therefore corresponds with expected degree of fold disruption.

We selected recombination events from our FULL and FIT datasets for which recombination breakpoints occurred in genome regions encoding the portions of Rep and CP with known/approximated 3-D structures. These events constituted a “real event” dataset.

We then used the permutation test devised by [11] to determine whether predicted CP and Rep fold disruptions incurred by real events were less severe than those incurred by random TYX-TOX recombination events simulated throughout the *rep* and *cp* genes. A set of simulated recombination events was produced, for each real event, by moving the breakpoints observed in the real event backwards and forwards along the entire nucleotide sequence alignment one non-synonymous nucleotide polymorphism position at a time until we produced every possible unique recombination event involving the “exchange” of exactly the same number of non-synonymous nucleotide polymorphisms as the corresponding real event. In cases where, for an individual real recombinant, more than two breakpoints were detected within the analysed region, the exact same procedure was followed as that mentioned above except that the entire set of breakpoints found in the real event were moved backwards and forwards across the sequences to produce simulated recombinants that resembled the real ones with respect to the numbers of non-synonymous nucleotide polymorphisms exchanged during recombination (see Figure S2 from [11] for simulation details). This set of simulated events was used to determine whether, given “exchanges” of the same numbers of polymorphic amino acids as were observed for real events, there was a significant tendency for the real recombination events to involve the transfer of less disruptive amino acid polymorphisms.

Quantification of potential fold disruption in real and simulated chimaeric CP and Rep molecules was carried out using the SCHEMA method implemented in RDP3 [12]. We summed the E scores determined for the observed chimeras and then determined 10 000 summed E-scores for chimaera sets where each of the observed chimeras was randomly replaced by a simulated chimaera that resembled it with respect to (1) the numbers of non-synonymous nucleotide polymorphisms derived from TYX and TOX and (2) numbers of recombination breakpoints. The proportion of simulated chimaera sets with summed E scores lower than or equal to that of the observed chimaera set is equivalent to the probability that the breakpoint distributions in the observed chimeras have not tended to avoid disruption of protein folding.

**ssDNA folding disruption tests**

In the same way that recombination has the potential to disrupt protein folding [10], it could potentially disrupt ssDNA folding. We used two separate permutation tests of ssDNA folding disruption to determine whether there was any evidence of recombinant sequences displaying significantly lower degrees of estimated ssDNA folding perturbation than that observed in randomly generated recombinant sequences. In the first test we considered the over-all MFE of folded genomes, and we therefore calculated the difference between the MFE estimates of the parental sequences with each of the real recombinants. We took the lower of the two calculated values and summed these for the recombinants to obtain an over-all estimate of the difference in MFEs (deltaMFE) between the parental and recombinant sequences. We then recalculated these deltaMFE estimates for 10^6^ permuted datasets. In each permuted dataset rather than using the actual recombinants, MFE estimates were randomly drawn from those determined for the 100 simulated counterparts of each of the real recombinants. The probability that the real recombinants displayed less ssDNA folding disruption than the simulated recombinants was taken to be the proportion of permuted datasets in which deltaMFE estimates were lower than or equal to those estimated for the real datasets.

The second test that we performed was an exact analogue of the SCHEMA based test described above for detecting protein folding disruption except that rather than considering amino acid-amino acid interactions inferred from known high resolution protein atomic models, the test considered predicted base-pairing interactions within computationally folded nucleotide sequences. We compared the predicted TYX and TOX ssDNA folds with those of each of the 50 unique recombinants from our experiment and counted for each recombinant the number of times: (1) nucleotides inferred not to be paired in both the TYX and TOX genomes in any of the predicted near MFE folds but which were inferred to be paired in any one of the recombinant’s predicted near MFE folds (a number referred to as “a” i.e. enumerating potentially *aberrant* ssDNA structures absent in the parental sequences that are essentially generated by recombination); (2) nucleotides inferred to be paired in both the TYX and TOX genomes in any one of the predicted near MFE folds of each but which were not inferred to be paired in any of the recombinant’s predicted near MFE folds (a number referred to as “b”; i.e. enumerating conserved ssDNA structures potentially present in parental sequences that get *broken* by recombination). We then redid these counts for each of the 100 simulated recombinants corresponding to each of the 50 actual recombinants (a total of 5000 simulated recombinants considered; see above). For the 50 real recombinants we summed all of the “a” counts and all of the “b” counts to respectively obtain the total values, T_a_ and T_b_, of these. We then recalculated these values for 10^6^ permuted datasets but, rather than using the actual recombinants in these calculations, for each permuted dataset, “a” and “b” values were randomly drawn from those determined for the 100 simulated counterparts of each of the real recombinants. The probability that the real recombinants displayed either less aberrant base-pairing (i.e. lower T_a_ scores) or fewer base-pairing disruptions (i.e. lower T_b_ scores) than the simulated recombinants was respectively taken to be the proportion of permuted datasets in which T_a_ and T_b_ scores were lower than or equal to those estimated for the real datasets.

**References**

1. Draghici H-K, Varrelmann M (2010) Evidence for similarity-assisted recombination and predicted stem-loop structure determinant in potato virus X RNA recombination. The Journal of general virology 91: 552-62.

2. Nagy PD, Bujarski JJ (1995) Efficient system of homologous RNA recombination in brome mosaic virus: sequence and structure requirements and accuracy of crossovers. Journal of virology 69: 131-40.

3. Baird HA, Galetto R, Gao Y, Simon-Loriere E, Abreha M, Archer J, Fan J, Rpbertson DL, Arts EJ, Negroni M (2006) Sequence determinants of breakpoint location during HIV-1 intersubtype recombination. Nucleic acids research 34: 5203-16.

4. Archer J, Pinney JW, Fan J, Simon-Loriere E, Arts EJ, Negroni M, robertson DL (2008) Identifying the important HIV-1 recombination breakpoints. PLoS computational biology 4: e1000178.

5. Zhang J, Temin HM (1994) Retrovirus recombination depends on the length of sequence identity and is not error prone. Journal of Virology 68: 2409-14.

6. Simon-Loriere E, Martin DP, Weeks KM, Negroni M (2010) RNA structures facilitate recombination-mediated gene swapping in HIV-1. Journal of Virology 84: 12675-82.

7. García-Andrés S, Tomás DM, Sánchez-Campos S, Navas-Castillo J, Moriones E (2007) Frequent occurrence of recombinants in mixed infections of tomato yellow leaf curl disease-associated begomoviruses. Virology 365: 210-9.

8. Lefeuvre P, Lett J-M, Varsani A, Martin DP (2009) Widely conserved recombination patterns among single-stranded DNA viruses. Journal of virology 83: 2697-707.

9. Heath L, van der Walt E, Varsani A, Martin DP (2006) Recombination patterns in aphthoviruses mirror those found in other picornaviruses. Journal of virology 80: 11827-32.

10. Voigt CA, Martinez C, Wang Z-G, Mayo SL, Arnold FH (2002) Protein building blocks preserved by recombination. Nature structural biology 9: 553-8.

11. Lefeuvre P, Lett J-M, Reynaud B, Martin DP (2007) Avoidance of protein fold disruption in natural virus recombinants. PLoS pathogens 3: e181.

12. Martin D, Lemey P, Lott M, Moulton V, Posada D, Lefeuvre P (2010) RDP3: a flexible and fast computer program for analyzing recombination. Bioinformatics 26: 2462-3.
