# Supplementary material for: Complex Recombination Patterns Arising during Geminivirus Coinfections Preserve and Demarcate Biologically Important Intra-Genome Interaction Networks
Source: PLoS Pathog. 2011 Sep 15;7(9):e1002203. doi: 10.1371/journal.ppat.1002203 (PMC3174254; doi:10.1371/journal.ppat.1002203)
Supplement: Table S1 — Summary of full genome sequences examined in this study. (DOC) [file ppat.1002203.s004.doc]

| **Table S1: Summary of full genome sequences examined in this study** | | | | |
| --- | --- | --- | --- | --- |
| Plant | Number of Sequences | Number of Recombinants | Distance to TYX* | Distance to TOX* |
| 1 | 53 | 12 | **0.06** | 0.12 |
| 2 | 18 | 9 | 0.14 | **0.03** |
| 3 | 103 | 3 | **0.03** | 0.15 |
| 4 | 15 | 4 | **0.06** | 0.12 |
| 5 | 16 | 14 | **0.03** | 0.15 |
| 6 | 7 | 6 | **0.08** | 0.10 |
| 7 | 60 | 16 | 0.09 | 0.09 |
| 8 | 25 | 8 | 0.14 | **0.04** |
| 9 | 32 | 10 | **0.07** | 0.11 |
| 10 | 33 | 24 | 0.14 | **0.04** |
| total | 362 | 106 | 0.10 | 0.08 |
| ^*^Raw distance including gaps, lower distance is in bold | | | |  |
