# Supplementary material for: Complex Recombination Patterns Arising during Geminivirus Coinfections Preserve and Demarcate Biologically Important Intra-Genome Interaction Networks
Source: PLoS Pathog. 2011 Sep 15;7(9):e1002203. doi: 10.1371/journal.ppat.1002203 (PMC3174254; doi:10.1371/journal.ppat.1002203)
Supplement: Table S2 — Testing for an association between predicted genomic secondary structures and recombination breakpoint positions. (DOC) [file ppat.1002203.s005.doc]

| **Table S2: Testing for an association between predicted genomic secondary structures and recombination breakpoint positions.** | |
| --- | --- |
| Tested structures | P-value |
| Structures predicted in TYX **OR** TOX | 0.8649 |
| Structures predicted in TYX **NOT** TOX | 0.1633 |
| Structures predicted in TOX **NOT** TYX | 0.0579 |
